# Do PD-1 and PD-L2 expressions have prognostic impact in hematologic malignancies?

**DOI:** 10.3906/sag-1706-194

**Published:** 2019-02-11

**Authors:** Serdal KORKMAZ, Selahattin ERDEM, Ebru AKAY, Erdem Arzu TAŞDEMİR, Hatice KARAMAN, Muzaffer KEKLİK

**Affiliations:** 1 Department of Hematology, Kayseri Training and Research Hospital, Kayseri Turkey; 2 Department of Internal Medicine, Kayseri Training and Research Hospital, Kayseri Turkey; 3 Department of Pathology, Kayseri Training and Research Hospital, Kayseri Turkey

**Keywords:** PD-1, PD-L2, multiple myeloma, acute leukemia, chronic lymphocytic leukemia

## Abstract

**Background/aim:**

PD-1 (programmed death-1) is an immune checkpoint receptor that modulates T-cell activity in peripheral tissues via interaction with its ligands, PD-L1 (programmed death-ligand 1) and PD-L2 (programmed death-ligand 2). Tumor cells upregulate PD-L1 or PD-L2 to inhibit this T lymphocyte attack. Our goal was to determine the PD-1 and PD-L2 expression rates of various hematologic malignancies, and evaluate whether PD-1 and PD-L2 expressions have an impact on prognosis.

**Materials and methods:**

For this purpose, pretreatment bone marrow biopsy specimens of 83 patients [42 multiple myeloma (MM), 21 acute leukemia, and 20 chronic lymphocytic leukemia (CLL)] were stained with monoclonal antibody immunostains of PD-1 and PD-L2.

**Results:**

As a result, the overall expression rate of PD-1 was 26.2%, 4.8%, and 60% in patients with MM, acute leukemia, and CLL, respectively, whereas the PD-L2 expression rate was 61.9%, 14.3%, and 10% in patients with MM, acute leukemia, and CLL, respectively.

**Conclusion:**

Finally, we concluded that the role of the PD-1 pathway can be demonstrated by immunohistochemistry (IHC). Since we evaluated whether there is a correlation between the (IHC) results and survival of patients with MM, acute leukemia, and CLL, we could not demonstrate meaningful evidence that these markers have an impact on prognosis.

## 1. Introduction

The PD-1/PD-L1 (programmed death-1/programmed death-ligand 1) pathway has led to major breakthroughs in the cancer immunotherapy field. PD-1 is an immune checkpoint receptor that modulates T-cell activity in peripheral tissues via interaction with its ligands, PD-L1 and PD-L2 (programmed death-ligand 2). PD-1 is expressed on activated T cells, B cells, and myeloid cells. Binding of PD-1 to its ligands limits effector T-cell activity, and therefore regulates detrimental immune responses and prevents autoimmunity (1). Upon antigen recognition, activated T cells express PD-1 on their surface and produce interferons that lead to the expression of PD-L1 in multiple tissues, including cancer (2). In progress, PD-L1 induces a coinhibitory signal in activated T cells and promotes T-cell apoptosis, T-cell anergy and T-cell functional exhaustion (3,4). Less is known about PD-L2, which is expressed on dendritic cells, macrophages, mast cells, and B cells (5).

Expression of PD-L1 and PD-L2 has been identified both on tumor cells and within the tumor microenvironment. Various tumor types such as breast cancer, gastric cancer, melanoma, and nonsmall-cell lung cancer are able to express PD-L1 (6). In addition to this, hematologic malignancies, such as multiple myeloma (MM), acute leukemia and chronic lymphocytic leukemia (CLL), have been shown to express PD-L1 or PD-L2 to some degree (7–13). 

The role of PD-1 pathway has been extensively investigated in nonhematologic malignancies, and upon understanding the importance of this pathway, anti-PD-1 therapeutic strategies have been developed to treat solid malignancies. However, the exact role of this pathway is not known in hematologic disorders, and it is being newly analyzed. In the literature, there are a limited number of reports regarding the effectiveness of anti-PD-1 agents in hematologic malignancies, and it is expected to be a new area to be explored in the near future. Therefore, the goal of this study was to demonstrate the PD-1 and PD-L2 expression rate of various hematologic malignancies and to evaluate whether PD-1 and PD-L2 expressions have an impact on prognosis. For this purpose, the bone marrow biopsy specimens of 83 patients with MM, acute leukemia, and CLL were stained with monoclonal antibody immunostains of PD-1 and PD-L2. 

## 2. Materials and methods 

The study was conducted retrospectively in Kayseri Training and Research Hospital. The departments of Hematology and Pathology contributed to this study. The patients who were alive, or their relatives if they were dead, provided their written informed consent for the participation. The study was approved by the local Ethics Committee and was in accordance with the Declaration of Helsinki. 

A total of 83 patients with various hematologic malignancies were enrolled in the study. Medical records of patients diagnosed between January 2011 and January 2016 were collected, retrospectively. The diagnostic bone marrow biopsy specimens of 83 patients were found in the archive of pathology. Briefly, tissues were fixed in 10% buffered formalin and paraffin-embedded. One paraffin-embedded block tissue was selected for each case and was cut into 4-µm sections. Tissue sections were deparaffinized by xylene and rehydrated with ethanol. The sections were incubated with commercially available mouse antihuman antibodies of PD-1 (NAT 105) (Ventana, catalog number: 760-4895) and PD-L2 (Anti-NeuN antibody; 1B7, ty25 ab) (Catalog number: 21107). Immunohistochemical staining was examined by using the avidin-biotin-peroxidase method. 

Each specimen was evaluated independently by 2 pathologists using polarized light microscopy. For each case, the section with the highest percentage of tumor cells stained was used for analysis. Namely, besides intensity, the tumor infiltration pattern was also annotated. Positive and negative IHC controls were routinely used. PD-1 and PD-L2 staining, which were observed in membrane and/or cytoplasm of tumor cells and immune cells, were considered positive if ≥1% of tumor cells had cytoplasmic-membranous staining or any positive immune cells with an intensity of 2+ or 3+ (0: no staining; 1+: 1%–20% of tumor cells; 2+: 20%–50% of tumor cells; 3+: ≥50% of tumor staining) as reported. 

### 2.1. Statistical analysis

All statistical analyses were performed using SPSS version 21.0 (SPSS, Chicago, IL, USA). Descriptive statistics were calculated for each of the variables. Data were expressed as medians and percentages. Overall survival (OS) time was calculated from the date of diagnosis to the date of death or last follow-up. Distribution differences of clinical characteristics between groups were analyzed with Pearson’s chi-square and survival curves were estimated with the Kaplan–Meier method and the groups were compared using the log-rank test. All P-values were 2-sided, and values were regarded as statistically significant if P < 0.05. 

## 3. Results

A total of 83 cases [29 female (34.9%) and 54 male (65.1%)] with hematologic malignancies [42 (50.6%) MM, 21 (25.3%) acute leukemia, and 20 (24.1%) CLL] were evaluated. Of 21 patients with acute leukemia, 17 were diagnosed with acute myeloid leukemia (AML), and the remaining were Philadelphia negative acute lymphoblastic leukemia (ALL). The median ages of the patients with MM, acute leukemia, and CLL were 69.5 (49–101), 65.6 (17–94), and 66.7 (38–94) years, respectively. The laboratory and clinical characteristics of the patients are exhibited in Table 1. Autologous stem cell transplantation (ASCT) was performed in 18 (42.9%) of the MM patients. Allogeneic stem cell transplantation data of the study population were not obtained. Organomegaly and lymphadenopathy were present in 10 (50%) and 16 (80%) of the CLL patients, respectively. Hyperviscosity syndrome occurred in 3 of the leukemia, and 5 of the myeloma patients, so plasma exchange therapy was performed for these patients.

**Table 1:** The laboratory and clinical characteristics of the study population.

Characteristics	Multiple myeloma(n = 42)	Acute leukemia(n = 21)	CLL(n = 20)
Age*	69.5 (49–101)	65.6 (17–94)	66.7 (38–94)
Sex (male/female)	28/14	15/6	11/9
Hb (g/dL)*	9.7 (6.1–11.3)	8.7 (3.7–15.9)	11.5 (5.7–16.9)
WBC (x109/L)*	6.0 (2.2–11.8)	4.9 (0.8–120.3)	29.4 (10.1–67.9)
PLT (x109/L)*	210 (13–562)	44 (14–401)	154.5 (43–447)
Creatinine (mg/dL)*	1.67 (0.6–8.6)	0.96 (0.4–4.0)	–
LDH (IU/L)*	187.5 (103–1314)	366 (46–2032)	286.5 (139–966)
Total protein (g/dL)*	8.7 (5.8–13.5)	-	-
Albumin (g/dL)*	3.2 (1.3–4.4)	-	-
Uric acid (mg/dL)*	6.7 (2.2–14.4)	6.1 (2.5–13.7)	5.9 (2.1–9.8)
Calcium (mg/dL)*	9.2 (7.0–13.9)	8.9 (7.8–10.3)	9.1 (7.6–11.9)
ALT (IU/L)*	21 (13–37)	17 (7–40)	19 (11–25)
AST (IU/L)*	25 (14–63)	26 (11–89)	22 (17–28)
ESR (mm/h)*	61 (23–120)	45 (27–69)	23 (2–61)
Beta 2 microglobulin (mg/L)*	11.3 (2.5–34.9)	-	6.3 (1.7–16.8)
IgG (g/L)*	17.7 (1.93–48)	-	8.2 (4.8–12.6)
IgA (g/L)*	0.62 (0.21–1.48)	-	0.62 (0.32–2.13)
IgM (g/L)*	0.18 (0.12–2.0)	-	0.21 (0.12–1.9)
Bone marrow plasma cells (%)*	50 (20–95)	-	-
Bone marrow blast percentage (%)*	-	70 (20–90)	-
B symptoms+, n(%)	-	-	9 (45)
SIFE results - IgG Kappa, n (%) - IgA Lambda,n (%) - IgA Kappa,n (%) - Light chain, n (%)	25 (59.5) 7 (16.7) 3 (7.1) 7 (16.7)	-	-
ISS - Stage I, n (%) - Stage II, n (%) - Stage III, n (%)	2 (4.8) 9 (21.4) 31 (73.8)	-	-
Rai stage - 0, n (%) - I, n(%) - II, n (%) - III, n (%) - IV, n (%)	-	-	4 (20) 6 (30) 2 (10) 4 (20) 4 (20)
Dead /alive	26/16	13/8	8/12

* median (range) Hb: hemoglobin, WBC: white blood cell, PLT: platelets, LDH: lactate dehydrogenase, CLL: chronic lymphocytic leukemia, SIFE: serum immunofixation electrophoresis ALT: alanine aminotransferase, AST: aspartate aminotransferase ESR: erythrocyte Sedimentation Rate, ISS: international Staging System

**Figure 1:**
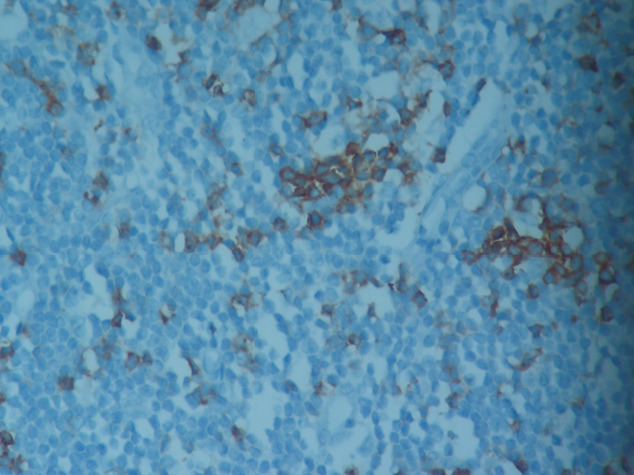
A representative image of a CLL patient showing 2+ cytoplasmic-membranous staining of PD-1 antibody (original magnification, 40×).

**Figure 2:**
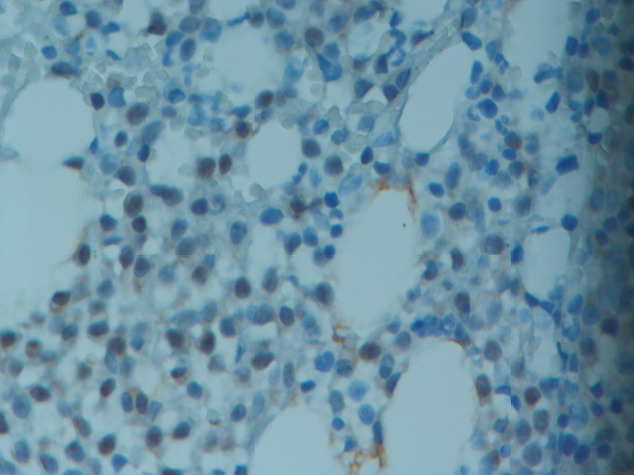
A representative image of a myeloma patient showing 1+ cytoplasmic-membranous staining of PD-L2 antibody (original magnification, 40×).

The overall expression rate of PD-1 was 26.2%, 4.8%, and 60% in patients with MM, acute leukemia, and CLL, respectively, whereas the PD-L2 expression rate was 61.9%, 14.3%, and 10% in patients with MM, acute leukemia, and CLL, respectively. Figures 1 and 2 are demonstrative examples showing immunostaining of PD-1 and PD-L2. A detailed summary of immunohistochemistry (IHC) stain results are summarized in Table 2. 

**Table 2:** Distribution of cases according to PD-1 and PD-L2 immunostains.

Hematologic malignancy	PD-1 expression 0 1 2 3	PD-L2 expression 0 1 2 3
MM (n = 42)	31 11 - -	16 18 8 -
AML (n = 17) ALL (n = 4)	16 1 - - 4 - - -	15 2 - - 3 1 - -
CLL (n = 20)	8 3 7 2	18 2 - -

MM: multiple myeloma, AML: acute myeloid leukemia, ALL: acute lymphoblastic leukemia, CLL: chronic lymphocytic leukemia, PD-1: programmed death-1, PD-L2: programmed death ligand 2

Of the patients, 16 out of 42 were alive in the MM group. Thirteen patients in the acute leukemia group and 8 patients in the CLL group had died of disease progression or unrelated causes. The median overall survival (OS) was 50.18 months (Hazard ratio (HR): 38.82–61.55; 95% CI), 54.57 months (HR: 40.73–68.41; 95% CI), and 40.85 months (HR: 29.55-52.15; 95% CI) in the MM, acute leukemia, and CLL groups, respectively. Since we have evaluated whether there is a correlation between IHC results and survival of patients, there was no significance between PD-1/PD-L2 expression rates and MM (P = 0.691/P = 0.546) and acute leukemia (P = 0.552/P = 0.273) and CLL (P = 0.319/P = 0.199) (Table 3). Also, we did not observe a correlation between PD-1/PD-L2 expression rates and International Staging System (ISS), and serum immunofixation electrophoresis (SIFE) results in the MM group. In addition, there was no correlation between PD-1/PD-L2 expression rates and disease stage and, B symptoms in the CLL. 

**Table 3:** The median overall survival (OS), 3 year OS, and 5 year OS of the study population; and the relationship between the median OS and expression rates of PD-1 and PD-L2.

	OS-3y (%)	OS-5y (%)	OS (months) Median (range)	PD-1+ (n, %)	PD-L2+ (n, %)	P-value	P-value
MM	60.4	54.4	50.18 (38.82–61.55)	11 (26.2)	26 (61.9)	P^1a^ = 0.691	p^1b^ = 0.546
AML/ALL	71.4	71.4	54.57 (40.73–68.41)	1 (4.8)	3 (14.3)	P^2a^ = 0.552	p^2b^ = 0.273
CLL	52.3	26.2	40.85 (29.55–52.15)	12 (60.0)	2 (10.0)	P^3a^ = 0.319	p^3b^ = 0.199

MM: multiple myeloma, AML: acute myeloid leukemia, ALL: acute lymphoblastic leukemia, CLL: chronic lymphocytic leukemia, PD-1: programmed death-1, PD-L2: programmed death ligand 2 P1a: Comparison of PD-1 expression rate and median survival in the MM group P1b: Comparison of PD-L2 expression rate and median survival in the MM group P2a: Comparison of PD-1 expression rate and median survival in the AML/ALL group P2b: Comparison of PD-L2 expression rate and median survival in the AML/ALL group P3a: Comparison of PD-1 expression rate and median survival in the CLL group P3b: Comparison of PD-L2 expression rate and median survival in the CLL group

## 4. Discussion

The PD-1 pathway plays a significant role in the regulation of T-cell activation and in apoptotic pathways of effector/memory T lymphocytes. The upregulation of PD-1 and PD-L1 may be a common phenomenon in hematologic malignancies. Data increasingly have shown that PD-1 is expressed at a higher level in T cells from tumor patients (14). Tumor cells upregulate PD-L1 or PD-L2 to inhibit this T lymphocyte attack. Binding of the PD-1 receptor with PD-L1 blocks phosphatidylinositol 3-kinase activation thus leading to downregulation of stimulatory proteins required for T-cell proliferation (15,16). The checkpoint inhibition by tumor cells via the PD-1 pathway suppresses the antitumor immune response. Tumor-associated immune suppression can lead to defective T-cell-mediated antitumor immunity and disease progression.

The role of PD-1 pathway has been extensively investigated in nonhematologic malignancies: however, it is not clear in hematologic malignancies. Expression of PD-L1 and PD-L2 has been identified both on tumor cells and within the tumor microenvironment. Data with evidence are limited in the literature. 

PD1 expression on T/NK cells and myeloma cells has been reported in some studies (17–19). Guo et al. showed a high level of PD-L1/PD-L2 expression on myeloma cell line RPMI 8226 (7). Sponaas et al. showed that none of the patients (n = 14) expressed PD-L2, but PD-L1 was found on the majority of myeloma cells (8). 

Salih et al. showed that the positive expression rate of PD-L1 in acute leukemia was 57% (9). However, Tamura et al. evaluated 30 samples of acute leukemia patients and did not find PD-L1 expression (20). In another study, of the 60 acute leukemia patients, 22 (36.7%) were positive for PD-L1 expression (54.3% were acute monocyte leukemia) while 38 (63.3%) were negative (10).

 PD-1 expression in CD4+ and CD8+ T cells was significantly higher in patients with CLL (21). Rusak et al. showed that CLL patients with advanced high-risk disease (stages III and IV) had a higher number of CD4+/PD1+ circulating T cells in peripheral blood compared with low-risk and intermediate-risk subjects (22). In CLL, CD8+ T cells expressed many immunosuppressive ‘exhaustion’ features including PD-1 and PD-L1 (11–13). 

In our study, the overall expression rate of PD-1 was 26.2%, 4.8%, and 60% in patients with MM, acute leukemia, and CLL, respectively. In addition, the overall expression rate of PD-L2 was 61.9%, 14.3%, and 10% in patients with MM, acute leukemia, and CLL, respectively. Therefore, the limited literature data and our results allow us to state that PD-1, PD-L1, and PD-L2 expression rates are highly heterogeneous.

The PD-1 pathway has been explored as a potential predictor of prognosis for hematologic malignancies. The associations of PD-1 or PD-L1 or PD-L2 expression and clinical outcomes have been variable across tumor subtypes. A study by Chen et al. concluded that PD-L1–negative patients had a better prognosis than the positive patients with acute leukemia (10). Rusak et al. concluded that treatment-naive patients with CLL with the number of CD4+/PD1+ T cells exceeding 15.79% at baseline showed a significantly shortened time to the first treatment compared with CLL patients with lower CD4+/PD1+ T cell numbers (6 months vs 18.5 months, respectively, P = 0.006) (22). Moreover, some other studies showed that the expansion of T cells expressing PD-1 correlated with an inferior outcome in CLL patients (11–13). However, Grzywnowicz et al. showed that expression of PD-1 and PD-L1 revealed no prognostic value in CLL patients (23). In our study, we have detected considerable PD-1 expression rates in patients with CLL, whereas PD-L2 expression rates were higher in patients with MM. Both PD-1 and PD-L2 expression rates were lower in acute leukemia. As a result, literature data regarding the association between survival and PD-1 and/or PD-L2 expression rates are very limited, and PD-1 and/or PD-L2 expression did not demonstrate a survival advantage or disadvantage in our study group. 

Blockade of the PD-1/PD-L1 pathway is a new and promising therapeutic approach in hematologic malignancies. Guo et al. demonstrated that PD-L1 and PD-L2 blocking on myeloma cells by the relevant blocking antibodies significantly improved the expanded NK cell cytotoxicity against myeloma cells in vitro (7). The use of anti-PD-1 therapy in hematologic malignancies is limited to early-phase clinical trials. Sponaas et al. proposed that MM patients may benefit from anti-PD-1/PD-L1 treatment (8). However, the use of PD-1 blockade in MM has been explored in several published clinical trials, with overall disappointing results. A phase I dose-escalating trial tested pidilizumab in 17 patients with refractory AML, CLL, Hodgkin, and non-Hodgkin lymphoma or MM and reported an acceptable safety profile, and clinical benefit in 33% of the patients evaluated (24). One recent phase I study in MM demonstrated that PD1 blocking by anti-PD1 antibody did not show significant treatment benefit (25). Another phase I trial of nivolumab in hematologic malignancies included 27 patients with relapsed/refractory MM (26). In this trial, patients were treated with nivolumab, and no objective responses observed, and the PFS at 24 weeks was 15% (26). There are multiple ongoing clinical trials for patients with MM, including trials of pembrolizumab monotherapy, the combination of pembrolizumab and lenalidomide, and the combination of pembrolizumab and pomalidomide, and the combination of pidilizumab. In addition, the initial small phase I trial of pidilizumab included seven patients with heavily pretreated AML (24). Patients were treated with various doses of pidilizumab, but only one patient showed clinical benefit, with minimal response, and the patient’s OS was 61 weeks (24). Additionally, clinical trials of nivolumab include monotherapy for AML is underway. Therefore, available data are not sufficient to conclude that blockade of the PD1/PDL1 pathway in these groups of patients is effective, so we have to wait of the future results of ongoing clinical trials.

This study has some limitations. First, this study is a single-center study with a relatively small sample size, which might underestimate or overestimate the results. Second, we performed IHC staining on pretreatment biopsy materials, so we do not know if PD-1 and/or PD-L2 expression rates might change after anti-PD-1 therapies. Third, because we could not provide the PD-L1 immunostaining, we could not evaluate the expression rate of PD-L1 in our specimens. 

In conclusion, IHC staining is a widespread method, easy to perform and cost-effective. We conclude that the role of PD-1 pathway can be demonstrated by IHC. Most of the IHC studies of PD-1, PD-L1, and PD-L2 are performed on cell lines in vitro, and also they are all limited in number. Furthermore, in the literature, it may be emphasized that the number of IHC studies in real life settings is very limited. In this context, we have made a study on patient bone marrow specimens showing the expression rates of PD-1 and PD-L2 in various hematologic malignancies; however, we could not find a meaningful evidence that these markers have an impact on prognosis. More specifically designed prospective studies are needed to externally cross-validate our findings in a larger cohort of patients. If IHC markers can be standardized in the future, especially a cutoff that defines a clinically significant positive and predictive value, it may help identify patients more likely to benefit from anti-PD-1 therapies. 

## Acknowledgments

This study was supported by Kayseri Training and Research Hospital’s general training budget (Decision date/number: 15.03.2016/51).
